# Periampullary Metastases from Breast Cancer: A Case Report and Literature Review

**DOI:** 10.1155/2019/3479568

**Published:** 2019-01-09

**Authors:** Yi Lin, Sio In Wong, Yuzhou Wang, Chileong Lam, Xianghong Peng

**Affiliations:** ^1^Department of Medical Oncology, Centro Hospitalar Conde de São Januário, Sé, Macau; ^2^Department of Pathology, Centro Hospitalar Conde de São Januário, Sé, Macau

## Abstract

We presented a metastatic breast cancer case who was afflicted with obstructive jaundice caused by an ampullary neoplasm. Since jaundice due to periampullary metastasis from breast cancer was a rare entity, a literature review of similar cases through the PubMed database was done. A total of 23 additional cases were found. Among these 24 cases, 5 presented with periampullary metastasis synchronously with the diagnosis of breast cancer, while 19 had metachronous periampullary metastasis with an interval ranging between 1.3 and 23 years from the initial diagnosis of breast cancer to the emergence of jaundice. It is intriguing to establish a differential diagnosis for common bile tract stricture prior to tissue biopsy, even with diagnostic workups including serum tumor markers, MRI plus MRCP, ERCP with intraductal brushing, and endoscopic ultrasound, in that the clinical, radiological, and endoscopic findings of metastatic lesions overlapped extensively with those found with primary periampullary malignancies. An immunohistochemical portfolio including cytokeratin7/20 (CK7/20), homeobox protein CDX2, human epidermal growth factor receptor 2 (HER2/neu), estrogen receptor alfa (ER*α*), progesterone receptor (PgR), mammaglobin, gross cystic disease fluid protein-15 (GCDFP-15), and transacting T-cell-specific transcription factor (GATA-3) was helpful for differential diagnosis among cases with ambiguous microscopic features.

## 1. Introduction

Obstructive jaundice caused by extrahepatic biliary tract metastases from breast cancer is a rare clinical scenario. Accurate and prompt differentiation between primary and secondary periampullary malignancies is essential for further treatment decision-making and will exert a major impact on the prognosis. We presented a case with breast cancer who developed metachronous metastasis to the ampulla of Vater while other distant metastatic lesions subsided completely after systemic treatment. A literature review through the PubMed database yielded a total of 23 similar cases of breast cancer with periampullary metastases. Differential diagnosis between periampullary metastasis from breast cancer and a primary periampullary cancer was discussed thoroughly regarding patient history, serum tumor markers, imaging study plus biopsy procedures, and histopathology.

## 2. Case Report

**Figure 1:**
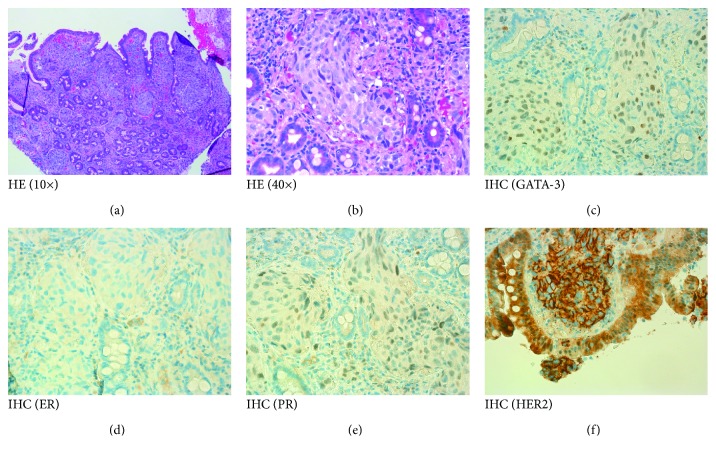
Histologic sections of the duodenal major papilla tumor biopsy. (a) Hematoxylin and eosin, 10× amplification. (b) Hematoxylin and eosin, 40× amplification. (c) GATA-3 (IHC), 40× amplification. (d) ER (IHC), 40× amplification. (e) PR (IHC), 40× amplification. (f) HER2 (IHC), 40× amplification. Isolated tumor cells and tumor cells clustered in solid pattern were seen in the lamina propria of the duodenal mucosa. The tumor cells have pleomorphic nuclei with prominent nucleoli. IHC profiling was weakly positive for GATA-3 (c), strongly positive for HER2 (f), and negative for ER (d) and PR (e).

A 42-year-old woman presented with right breast invasive ductal carcinoma (TNM stage: cT3N1M0) which was human epidermal growth factor receptor 2 (Her2) overexpressed and estrogen receptor (ER) and progesterone receptor (PR) negative in August 2013. Modified radical mastectomy was performed in November 2014 after finishing 8 cycles of preoperative chemotherapy with trastuzumab incorporated. The surgical specimen had resection margins clear of tumor cells and was staged as ypT2N2a. She was afflicted with right chest wall local recurrence less than two months after the mastectomy. Complete remission of the recurrence was achieved by local external beam irradiation. Administration of trastuzumab was continued to a total of one year. Nonetheless, seven months after completion of locoregional radiotherapy, some right chest wall skin lesions appeared in October 2015 with enlarged ipsilateral supraclavicular lymph nodes, which were both confirmed to be recurrent breast cancer by biopsy. She received salvage chemotherapy with paclitaxel plus pertuzumab and trastuzumab every 3 weeks. The disease progressed with multiple liver and lung metastases in April 2016. Ado-trastuzumab emtansine was administered every 3 weeks, and the metastatic lesions subsided completely on 2 serial contrast-enhanced CT scans in August and September 2016. Nonetheless, the patient was afflicted with rapidly worsening jaundice in late September 2016. Meanwhile, serial elevation of serum levels of carcinoembryonic antigen (CEA) was detected with fluctuating serum levels of carbohydrate antigen 15.3 (CA15.3) and carbohydrate antigen 19.9 (CA19.9). Magnetic resonance cholangiopancreatography (MRCP) showed segmental thickening of the common bile duct which was hypointense on T1WI and hyperintense on T2WI with contrast enhancement. A swollen, hyperemic major duodenal papilla and a well-demarcated luminal stricture 7 cm in length spanning the middle and lower portions of the common bile duct were detected in endoscopic retrograde cholangiopancreatography (ERCP). ERCP brushing cytology yielded suspicious malignant cells. Forceps biopsy from the major duodenal papilla was consistent with poorly differentiated adenocarcinoma (Figures 1(a) and 1(b)), of which histopathologic features showed no overt similarity to those of the prior mastectomy specimen. Immunohistochemistry (IHC) profiling was positive for cytokeratin7 (CK7), Her2, transacting T-cell-specific transcription factor GATA-3 (Figure 1(c)) and negative for cytokeratin20 (CK20), ER, PR, and gross cystic disease fluid protein-15 (GCDFP-15) (Figures 1(d)–1(f)). The ampullary lesion was considered to be a metastasis from breast cancer. The patient's jaundice exacerbated in spite of papillosphincterectomy and bile tract stenting. She deceased in December 2016.

## 3. Discussion

Although adenocarcinoma of the periampullary region consistently present with obstructive jaundice, this entity is composed of primary tumors derived from the pancreatic head, duodenum, distal biliary duct, and ampulla in addition to secondary deposits from distant sites including the lung, intestine, kidney, melanoma, and breasts.

Cases presenting with obstructive jaundice due to metastases to the periampullary region from breast cancer have been only occasionally reported in the literatures [1].

In order to summarize the cases presenting with obstructive jaundice due to periampullary metastasis from breast, key words including “breast cancer metastasis”, “secondary malignancies of/metastases to periampullary region/pancreatic head/ampulla of Vater/biliary tract/duodenum”, plus “malignant obstructive jaundice” were used to search the PubMed database for related literatures in English with full text available between 1995 and 2016. Cases included should have all of the following data: (1) pathologically confirmed primary breast cancer and secondary periampullary metastases involving ampulla of Vater, duodenum, pancreatic head, or extrahepatic biliary tract; and (2) tomography imaging studies such as CT, MR, or PET to evaluate metastasis to other parts of body. Metastatic breast cancer cases that had obstructive jaundice due to disseminated intrahepatic metastasis or extrahepatic biliary tract compression by enlarged peritoneal lymph nodes or biliary tract stricture of undetermined site were excluded since we intended only to include the cases that featured clinical characteristics indistinguishable from primary periampullary cancer. Supplementary Table 1 listed our case in addition to 23 cases found through literature review.

It has been reported that up to 21% of malignant extrahepatic biliary obstructions resulted from distant metastases [2]. To our knowledge, the differential diagnosis between breast cancer metastasis to the periampullary region and primary periampullary cancer has not been discussed thoroughly in the published articles yet. We hereby summarized the data and focused on the differential diagnosis with regard to patient history, serum tumor markers, imaging study plus biopsy procedures, and histopathology, etc.

### 3.1. History

Among the 24 cases in our review, 5 presented with periampullary lesions causing jaundice while breast cancer was detected concurrently by subsequent diagnostic workups (described as “synchronous” in column 3 in Supplementary Table 1). 16 cases developed metachronous periampullary metastasis as the first sign of recurrence after curative resection of breast cancer, with a recurrence-free interval ranging from 1.5 years to 23 years (described as “metachronous” in column 3 in Supplementary Table 1). The remaining 3 cases had other sites of distant metastasis from the breast prior to the emerging of periampullary metastasis (also described as “metachronous” in column 3 in Supplementary Table 1). As for these 3 cases, obstructive jaundice presented in a scenario that all the formerly detected distant metastasis subsided or remained stable during systemic treatment. Therefore, metastases should be taken into account for differential diagnosis among patients with distal biliary stricture who has prior history of breast cancer. Meanwhile, the possibility of a second primary periampullary malignancy in breast cancer survivors should also be considered. It has been reported that the standardized incidence ratio (SIR) estimates for second primary cancer risk after breast cancer were 1.51 (95% CI: 1.35–1.70) for women younger than 50 years and 1.11 (95% CI: 1.02–1.21) for those who were older [3]. A population-based case-control study also showed that breast cancer survivors were exposed to an excess risk of developing a second primary cancer, and the HER2-positive status increased cancer incidence risk of the digestive system and thyroid, while BRCA1 or BRCA2 mutation increased the cancer incidence risk of the genital system [4].

### 3.2. Serum Tumor Markers

Among the patients with available data in our review, elevated serum CEA level was detected in 4 out of 11 patients, elevated serum CA19.9 in 4 out of 10 patients, elevated serum CA15.3 in 6 out of 11 patients, and combination of elevated level of CA15.3 and normal level of CA19.9 in 1 out of 7 patients. Elevated serum CEA could be detected in gastrointestinal, pancreaticobiliary, lung, breast, medullary thyroid carcinoma, and multiple nonneoplastic conditions. CA19.9 is an established serum marker for the diagnosis of pancreaticobiliary carcinoma, with a sensitivity and a specificity of 76.7% and 87.1%, respectively, for pancreatic cancer, as well as 77.6% and 83% for biliary cancer without cholangitis or cholestasis at a cutoff value of 37 units/ml [5]. Elevated serum CA19.9 levels were also found in breast cancer patients with only axillary lymph nodes recurrence [6]. Therefore, the significance of serum levels of CEA and CA19.9 remain uncertain in the differentiation between periampullary breast cancer metastases and primary pancreatobiliary cancer among patients with extrahepatic biliary stricture. Moreover, there are no evidences that showed that the serum tumor marker CA 15.3 could be used for diagnosis of metastatic breast cancer [7].

### 3.3. Imaging/Endoscopic Diagnostic Workups and Procedures for Biopsy

Contrast-enhanced CT scan was usually the initial imaging study for patients presenting with painless jaundice. The majority of these 24 cases were found to have a mass in the head of the pancreas with or without involvement of the duodenum and common biliary tract on CT scan. Indirect signs of the biliary stricture as dilated proximal biliary tract were seen universally in all the cases, including those without overt periampullary mass on CT scan. It may be difficult to differentiate a solitary pancreatic metastasis from a primary pancreatic tumor only via CT scan. Metastatic masses may be hypo- or isoattenuating at nonenhanced CT, and their margins might be clearly demarcated, ill-defined, or lobulated. MRI scan usually showed tumor which was hypointense on T1-weighted images and had intermediate or high signal intensity on T2-weighted images. On contrast enhancement CT/MRI scan, primary pancreatic adenocarcinoma generally manifests as a hypoenhancing mass. In contrast, 3 cases with pancreatic metastases from invasive lobular breast cancer in a case report unanimously showed rim enhancement [8]. However, the enhancement pattern of hypovascular pancreatic metastases from the lung, breast, and colon may also resemble that of the primary pancreatic adenocarcinoma [9].

MRCP has a high sensitivity for detecting bile duct stenosis and filling defects associated with bile duct carcinoma. However, it cannot reliably distinguish malignant strictures from benign strictures nor differentiate between metastases and primary biliary malignancy [10].

Biopsy of the malignant biliary stricture is essential for diagnosis, which may be obtained by forceps biopsy, brush cytology, endoscopic ultrasound-guided fine-needle aspiration (EUS-FNA), and cholangioscopy-directed biopsy. Among the 24 cases summarized in our review, 19 (78.16%) underwent upper gastrointestinal endoscopy and 13 (54.17%) underwent ERCP. The neoplasms of the duodenum major papilla or ampulla were visualized endoscopically in 3 and 4 cases, respectively. Brush cytology is one of the most frequently used biopsy techniques with a sensitivity for diagnosing cholangiocarcinoma (23 to 80%) higher than that for pancreatic cancer (0 to 66%) [11]. ERCP forceps biopsy can provide a sample deep into the epithelium and theoretically avoids inadequate sampling that may occur with brushing [12]. 5 of the 24 patients underwent ERCP forceps biopsy, whereas it failed to obtain diagnostic specimen for only 1 case with the target lesion located in the head of pancreas. A recent study showed that the sensitivity of forceps biopsies for malignant biliary strictures was about 73.53% in cholangiocarcinoma, 29.17% for pancreatic head cancer, and 42.86% for other etiologies (metastasis from colon cancer, hepatocellular carcinoma, gallbladder cancer, and lung cancer) [13].

Endoscopic ultrasound-guided fine-needle biopsy (EUS-FNA) was performed in 3 patients with lesion in the head of the pancreas. Adequate diagnostic specimens were obtained for all of them. It has been reported that the sensitivity for diagnosing primary biliary duct malignancy via EUS-FNA was 43 to 86% [14–16], while the sensitivity and specificity for diagnosing pancreatic metastases via EUS-FNA was 75% to 93.8% and 60% to 100%, respectively [17, 18].

### 3.4. Histopathology and Immunohistochemistry Profile

Microscopic histopathology features resembling those of primary breast cancer in the periampullary specimen were considered to be an essential clue for diagnosis in 17 among these 24 cases. The metastasis usually featured disaggregated tumor cells or tumor cells in single file pattern in the pancreaticobiliary parenchyma. The primary breast lesion was lobular carcinoma in 11 of the aforementioned 17 cases. Invasive ductal carcinomas and invasive lobular carcinoma, as the most prevalent two types, account for 50–80% and 5–15% of all the breast cancer cases, respectively [19]. Infiltrating lobular carcinoma seems to have a metastatic pattern distinct from that of the ductal type, with an apparent predilection for the gastrointestinal tract due to unidentified mechanism [20].

IHC played an important role in differential diagnosis. The CK7+ve/CK 20-ve phenotype was found in both lobular and ductal breast carcinoma, while the CK7+ve/CK 20-ve or CK7+ve/CK 20+ve could be observed in primary biliary tract, ampulla, duodenal, or pancreatic carcinoma [21]. Among 4 of these 24 patients having both CK7 and CK20 IHC staining data, 3 were found to have CK7+ve/CK 20-ve phenotype, the remaining 1 had CK7-ve/CK 20-ve phenotype. Since CK 20 was usually not observed in metastatic breast cancer, it can be used to rule out metastatic breast carcinoma. Moreover, homeobox protein CDX2 expression was considered specific for enterocytes and was found in 97% of colorectal cancer, 61% of gastric cancer, and 16% of pancreatic cancer, whereas its expression in breast cancer has never been reported [22].

Approximately 75% to 80% of human breast tumors express ER and/or PR [23]. Amplification of HER2 gene or overexpression of Her2 protein was detected in 18% to 20% of human breast cancers [24]. ER/PR and Her2 status may show significant discordance between primary breast lesion and metastatic sites [25]. Changes in ER, PR, and HER2 status have also been observed in a large number of patients over the course of disease progression [26]. On the other hand, expressions of ER*α* and ER*β* were also detected in a variety of nonbreast cancers including gastric cancer [27], cholangiocarcinoma [28], gallbladder cancer [29], and pancreatic cancer [30]. Since Her2 overexpression was also reported in pancreaticobiliary tract cancer [31] and periampullary carcinoma [32], the potency of Her2 status for differentiating between primary periampullary cancer and metastatic breast cancer was diminished.

Mammaglobin was reported to be expressed in 70% to 80% of primary and metastatic breast tumors with the expression level unaltered at the metastatic site in comparison with the primary tumor, which made it a useful marker for identifying breast carcinoma especially localized in rare metastatic sites [33]. GCDFP-15 is a major protein constituent of breast cysts, of which expression was found in breast cancer and other cancers originating from nonmammary tissues such as the skin, salivary gland, bronchial gland, prostate, and seminal vesicle [34]. The expressions of the two human milk fat globule membrane protein epitopes, HMFG1 and HMFG2, were found in the lactating human mammary epithelial cells, as well as in neoplasms derived from the breast and ovary [35]. The combination of HMFG with GCDFP-15 was used to confirm the breast origin of the pancreatic lesion in 1 case [36]. GATA-3 was reported to be expressed in primary and metastatic breast ductal and lobular carcinoma (>90%), pancreatic ductal carcinoma (37%), gastric, and colon adenocarcinoma (<10%), which may be used to differentiate the tumor origin in combination with other related IHC markers [37].

### 3.5. Treatment and Prognosis

Jaundice in patients with breast carcinoma is usually attributed to extensive hepatic metastases and is associated with poor prognosis. However, the overall survival in patients who had extrahepatic metastatic obstruction without liver parenchymal involvement was significantly longer (median: 6 months) than that of patients with liver involvement (median: 1.8 months) [38].

10 out of 13 cases with a solitary periampullary metastasis in this series underwent radical resection of metastasis and achieved postoperative survival of 5 months to more than 48 months (median: 15 months). As for the other 11 cases with metastases other than the periampullary lesion, survival ranged between 0.5 and 54 months (median: 12 months) from the interventional procedure to alleviate jaundice Although pancreaticoduodenectomy is the treatment of choice for primary periampullary malignancies, the benefit of such treatment to the breast cancer patients with periampullary metastasis remains unclear.

## 4. Conclusion

It is quite challenging to differentiate between primary and secondary periampullary malignancies, especially for breast cancer patients with periampullary metastasis, in that the clinical, radiological, and endoscopic findings of metastatic lesions overlapped extensively with those found with primary periampullary malignancies. Histopathological features of the periampullary specimen similar to those observed in breast specimen were important clues for diagnosis. Immunohistochemistry profiling data including CK7/CK20, CDX2, ER, PR, Her2, mammaglobin, GCDFP-15, GATA-3 also plays an important role in differential diagnosis.

### 4.1. Clinical Practice Points

- Obstructive jaundice caused by extrahepatic biliary tract metastases from breast cancer is rare
- It is quite challenging to differentiate between primary and secondary periampullary malignancies in that the clinical, radiological, and endoscopic findings of metastatic lesions overlapped extensively with those of primary periampullary malignancies
- Histopathological examination of the periampullary specimen was essential for diagnosis. Immunohistochemistry profiling including CK7/20, CDX2, HER2, ER, PR, GCDFP-15, and GATA-3 plays an important role in differential diagnosis

## Supplementary Materials

Supplementary Materials Supplementary Table 1. List of our case in addition to the 23 similar cases we have retrieved in the PubMed database. The content of this table included clinical presentation, serum tumor markers, biopsy procedures, histopathology subtype plus immunohistopathology profile, and corresponding citations of each individual case.
