# Supplementary material for: Periampullary Metastases from Breast Cancer: A Case Report and Literature Review
Source: Case Rep Oncol Med. 2019 Jan 9;2019:3479568. doi: 10.1155/2019/3479568 (PMC6343154; doi:10.1155/2019/3479568)
Supplement: Supplementary Materials — Supplementary Table 1. List of our case in addition to the 23 similar cases we have retrieved in the PubMed database. The content of this table included clinical presentation, serum tumor markers, biopsy procedures, histopathology subtype plus immunohistopathology profile, and corresponding citations of each individual case. [file 3479568.f1.docx]

Supplementary Table 1: The 24 cases with periampullary metastasis from breast cancer

| No. | Age of diagnosis of breast cancer (years) | Emerging of periampullary metastasis relative to the diagnosis of breast cancer (interval in years) | Sites of lesion causing jaundice | Other sites of metastases emerging before (underlined) or concurrently with jaundice | Proocedures to obtain periampullary specimen, with the one yielding malignancy underlined | Histopathology of breast | Serum level of tumor markers | Hormone receptor status | | HER2 status | | Morphological features resembling those of primary breast cancer/IHC staining other than ER, PgR, Her2 in the periampullary specimen | Systemic treatment after jaundice | Overall survival from onset of jaundice to last follow-up (Months) | Citation |
| --- | --- | --- | --- | --- | --- | --- | --- | --- | --- | --- | --- | --- | --- | --- | --- |
|  |  |  |  |  |  |  |  | breast | Peri-ampulla | Breast  (overexpression/amplification) | Peri-ampulla |  |  |  |  |
| 0 | 46 | Metachronous (2.3)* | ampulla | Soft tissue, limited lung and liver | ERCP forceps biopsy | IDC | CEA/CA19.9/CA15.3 high | ER-ve, PgR+ve | ER-ve, PgR-ve | +ve | +ve | No/ CK7 +ve, CK19 +ve, CK20 -ve, GCDFP-15 -ve | None | 3 | Our case |
| 1 | 59 | Synchronous& | ampulla | Bone | ERCP forceps biopsy | IDC | CEA/CA19-9 normal | NM | NM | NM | NM | Yes/NM | Ct + Tras | 12 | Giestas S. 2016 [1] |
| 2 | 47 | Metachronous (23) | Common bile duct | Soft tissue | EUS- FNA | IDC | NM | ER+ve, PgR+ve | ER+ve, PgR+ve | -ve | -ve | NM/NM | Ct,Et | >12 | \| Cochrane J. 2015 \| \| --- \|   [2] |
| 3 | 44 | Synchronous | Pancreatic head | none | ERCP brushing, EUS-FNA followed by PDD | IDC | CA15.3 high, CA19.9/CEA normal | ER-ve, PgR-ve | ER-ve, PgR-ve | -ve | -ve | No/ Ck7, CK20 -ve. CK19, mammaglobin +ve | Ct | >8 | Tunio MA.2015 [3] |
| 4 | 43 | Metachronous (6) | Common bile duct and pancreas | Supraclavicular, mediastinal, intraperitoneal lymphadenopathy | ERCP brushing + excision of hepatoduodenal ligament lymph nodes | IDC | NM | ER+ve, PgR+ve | ER+ve, PgR+ve | -ve | -ve | Yes/NM | Et | >24 | Budimir I. 2015 [4] |
| 5 | 68 | Synchronous | Ampulla + pancreatic head | none | ERCP brushing followed by PDD | ILC | CA15.3/CA19.9 high  CEA normal | ER+ve, PgR+ve | ER+ve, PgR+ve | -ve | -ve | Yes/ Mammaglobin +ve | Et | >12 | Molino C. 2014 [5] |
| 6 | 56 | Metachronous (13) | Common bile duct, extraluminal | none | ERCP brushing followed by bile duct resection+ hepaticojejunostomy | IDC | CEA/CA19.9/CA15.3 normal | ER+ve, PgR+ve | ER+ve, PgR+ve | -ve | -ve | No/CK7+ve, CK20-ve, mammaglobin +ve | Et | >24 | Coletta M. 2014 [6] |
| 7 | 58 | Metachronous (18) | Pancreatic head, duodenum | None | ERCP brushing/forceps biopsy/PDD | ILC | NM | ER+ve, PgR+ve | ER+ve, PgR+ve | -ve | -ve | Yes/NM | Et | >48 | Bednar F. 2013[7] |
| 8 | 59 | Metachronous (15) | Pancreatic head | Bone, liver | EUS-FNA | IDC/ILCmixed | NM | ER+ve, PgR+ve | ER+ve, PgR+ve | -ve | -ve | NM/NM | Ct/Et | >13 | PAN B. 2012 [8] |
| 9 | 51 | Metachronous (1.6) | Pancreatic head | None | PDD | ILC | CEA/CA19.9/CA15.3 normal | ER-ve, PgR-ve | ER+ve, PgR+ve | -ve | +ve (IHC 3+) | Yes/NM | Ct, Et | 37 | Bonapasta SA. 2010 [9] |
| 10 | 39 | Synchronous | ampulla | none | ERCP brushing + forceps biopsy | ILC | NM | NM | NM | NM | NM | Yes/CK-AE1/3 +ve | None | NM | Rego RF 2009 [10] |
| 11 | 66 | Metachronous (2) | ampulla | none | ERCP brushing + forceps biopsy | IDC | NM | NM | NM | NM | NM | Yes/CK7+ve, | Ct | NM | Rego RF 2009 [10] |
| 12 | 57 | Metachronous (1.5) | Ampulla, Duodenum | Peritoneal dissemination | UGIE biopsy followed by PDD | ILC | CEA high， CA19.9 normal | ER+ve, PgR+ve | ER-ve, PgR-ve | -ve | NM | Yes/CK7+ve, CK20 -ve, | Ct, Et | 10 | Nihon-Yanagi Y.2009 [11] |
| 13 | 58 | Metachronous (5) | Duodenum bulb and pancreatic head | Right adrenal glands, abdominal lymphadenopathy | UGIE biopsy | IDC | CEA， CA19.9 high, CA15.3 normal | ER+ve, | ER+ve, | NM | NM | Yes/NM | Ct | >12, | Wang X. 2009 [12] |
| 14 | 60 | Synchronous | Duodenum, pancreatic head, CBD | bone | UGIE, ERCP brushing cytology, followed by PDD | ILC | CA15.3, CA19.9 high, | ER+ve, PgR+ve | ER+ve, PgR+ve | -ve | -ve | Yes/NM | Ct | >2, | Pérez Ochoa A. 2007 [13] |
| 15 | 50 | Metachronous (12) | Distal bile duct + pancreatic head | none | PDD | ILC | NM | ER+ve, | ER+ve, | -ve | -ve | Yes/NM | None | >12 | Stoeckler F. 2007 [14] |
| 16 | 71 | Metachronous (14) | pancreatic head | lungs | Laparotomy biopsy+palliative bypass | ILC | NM | ER+ve, | ER+ve, | -ve | -ve | Yes/NM | Ct | NM | Haque S. 2005[15] |
| 17 | 67 | Metachronous (3) | pancreatic head, ampulla of vater | none | PDD | ILC | CEA, CA19.9, CA15.3 normal | ER+ve, | ER+ve, | Ukn | Ukn | Yes/NM | Et | >37 | Crippa S. 2004[16] |
| 18 | 46 | Metachronous (9) | pancreatic head | Bones, brain | PTCD, autopsy | IDC | CA15.3, CEA high | ER+ve,  PgR+ve | ER-ve, PgR-ve | Ukn | Ukn | Yes/HMFG1 and 2, GCDFP-15 +ve, PAS -ve | none | 0.5 | Kitamura N.2003[17] |
| 19 | 40 | Metachronous (6.8) | duodenal papilla, pancreatic head | none | PTCD, UGIE biopsy, followed by PDD | ILC | CA15.3, CEA normal | ER-ve, PgR+ve | NM | NM | NM | Yes/CA153 +ve | Ct, Et | >18 | Nomizu T. 1999[18] |
| 20 | NM | Metachronous (8) | pancreatic head | Omental carcinomatosis | ERCP,  Laparotomy biopsy+palliative bypass | ILC | NM | NM | NM | NM | NM | NM/NM | Ct | 54 | Z’graggen K. 1998 [19] |
| 21 | 57 | Metachronous (1.3) | pancreatic head | none | ERCP, Laparotomy biopsy+palliative bypass | ILC | CA15.3 high, CEA normal | ER+ve, | ER+ve | NM | NM | Yes/NM | Et | >24 | Mountney J. 1997 [20] |
| 22 | 46 | Metachronous (4) | ampulla of vater, Common bile duct | none | ERCP brushing cytology, PDD | IDC | NM | ER+ve,  PgR+ve | ER+ve,  PgR+ve | NM | NM | No/NM | Ct | >4 | Titus AS. 1997 [21] |
| 23 | 48 | Metachronous (10) | Bifurcation of the common bile duct | None | ERCP (failed),  PTCD, choledochoduodenostomy+cholesystectomy | NM | NM | ER-ve,  PgR-ve | ER-ve,  PgR-ve | NM | NM | Yes/NM | none | 5 | Papo M. 1996 [22] |

Abbreviation: CA19-9= carbohydrate antigen 19-9; CA125= carbohydrate antigen 125; CA153= carbohydrate antigen 153; CBD= common bile duct; CEA= carcinoembryonic antigen; CK= cytokeratin; Ct= Chemotherapy; ERCP= Endoscopic retrograde cholangiopancreatography; ER= estrogen receptor; ET= endocrine therapy; EUS+FNA = Endoscopic ultrasound + fine needle biopsy; HMFG= human milk fat globule; IDC= invasive ductal carcinoma, ILC= invasive lobular carcinoma, IHC= immunohistochemistry; NM= not mentioned; PDD=Pancreaticoduodenectomy; PgR= Progesterone receptor; PTCD= percutaneous transhepatic cholangio-drainage; Tras= Trastuzumab; Ukn= unknown; UGIE= upper gastrointestinal endoscopy; +ve=positive; -ve=negative. NOTE: *: “Metachronous” means that there is an interval between the emerging of periampullary metastasis and the diagnosis of breast cancer. &:“synchronous” means that the cases presented with periampullary lesions causing jaundice while primary breast cancer was detected concurrently by subsequent diagnostic workups.

**Citations**

| \| 1. Giestas S, Lopes S, Souto P, et al. Ampullary Metastasis from Breast Cancer: A Rare Cause of Obstructive Jaundice. GE Port J Gastroenterol. 2016; 23(6):300-303.  2.Cochrane J, Schlepp G. Metastatic Breast Cancer to the Common Bile Duct Presenting as Obstructive Jaundice. Case Rep Gastroenterol. 2015; 9(2):278–284.  3. Tunio MA, Fatani H, Riaz K, et al. Pancreatic metastasis of breast cancer: a rare cause of obstructive jaundice. J Dow Uni Health Sci 2015; 9(1):1-2.  4. Budimir I, Pusic MS, Nikolic M, et al. Obstructive Jaundice as an Uncommon Manifestation of Metastatic Breast Cancer. World J Oncol. 2015; 6(1):297-300.  5.[Molino](https://www.ncbi.nlm.nih.gov/pubmed/?term=Molino%20C%5BAuthor%5D&cauthor=true&cauthor_uid=24387226)C,[Mocerino](https://www.ncbi.nlm.nih.gov/pubmed/?term=Mocerino%20C%5BAuthor%5D&cauthor=true&cauthor_uid=24387226) C, [Braucci](https://www.ncbi.nlm.nih.gov/pubmed/?term=Braucci%20A%5BAuthor%5D&cauthor=true&cauthor_uid=24387226) A, et al. Pancreatic solitary and synchronous metastasis from breast cancer: a case report and systematic review of controversies in diagnosis and treatment. World J Surg Oncol. 2014; 12:2  6.[Coletta](https://www.ncbi.nlm.nih.gov/pubmed/?term=Coletta%20M%5BAuthor%5D&cauthor=true&cauthor_uid=25515643) M, [Montalti](https://www.ncbi.nlm.nih.gov/pubmed/?term=Montalti%20R%5BAuthor%5D&cauthor=true&cauthor_uid=25515643) R, [Pistelli](https://www.ncbi.nlm.nih.gov/pubmed/?term=Pistelli%20M%5BAuthor%5D&cauthor=true&cauthor_uid=25515643) M, et al. Metastatic breast cancer mimicking a hilar cholangiocarcinoma: case report and review of the literature. World Journal of Surgical Oncology. 2014; 12:384.  7. Bednar F, Scheiman JM, McKenna BJ, et al. Breast cancer metastases to the pancreas. J Gastrointest Surg. 2013; 17(10):1826–1831.  8. [Pan B](https://www.ncbi.nlm.nih.gov/pubmed/?term=Pan%20B%5BAuthor%5D&cauthor=true&cauthor_uid=22493384), [Lee Y](https://www.ncbi.nlm.nih.gov/pubmed/?term=Lee%20Y%5BAuthor%5D&cauthor=true&cauthor_uid=22493384), [Rodriguez T](https://www.ncbi.nlm.nih.gov/pubmed/?term=Rodriguez%20T%5BAuthor%5D&cauthor=true&cauthor_uid=22493384), et al. Secondary Tumors of the Pancreas: A Case Series. Anticancer Res. 2012; 32(4): 1449-1452.  9. Bonapasta SA, Gregori M, Lanza R, et al. Metastasis to the pancreas from breast cancer: difficulties in diagnosis and controversies in treatment. Breast Care (Basel). 2010; 5(3):170–173.  10. Rego RF, Atiq M, Velchala N,et al. Ampullary metastasis from breast cancer. Endoscopy. 2009; 41Suppl 2: E278–E279.  11. Nihon-Yanagi Y, Park Y, Ooshiro M. et al. A case of recurrent invasive lobular carcinoma of the breast found as metastasis to duodenum.Breast Cancer. 2009; 16(1): 83–87.  12. Wang X, Meng X, Zhang T, et al. A case of breast cancer found as metastasis to the duodenum. J Cancer Res Exp Oncol. 2009; 1(2): 012-014.  13. Pérez Ochoa A, Sáez Hernáez F, Cajigas Fernández C, et al. Pancreatic metastases from ductal and lobular carcinomas of the breast. Clin Transl Oncol. 2007; 9(9):603-605.  14. Stoeckler F, Hagmuller E, Rumpelt HJ, et al. A rare cause of distal bile duct stenosis. J Gastrointest Cancer. 2007; 38(2-4):157–159.  15.[Haque S](https://www.ncbi.nlm.nih.gov/pubmed/?term=Haque%20S%5BAuthor%5D&cauthor=true&cauthor_uid=16447487), [Gopaldas RR](https://www.ncbi.nlm.nih.gov/pubmed/?term=Gopaldas%20RR%5BAuthor%5D&cauthor=true&cauthor_uid=16447487), [Plymyer MR](https://www.ncbi.nlm.nih.gov/pubmed/?term=Plymyer%20MR%5BAuthor%5D&cauthor=true&cauthor_uid=16447487), et al. Pancreatic mass of unusual etiology: case report of metastatic disease after a prolonged lag phase. [Am Surg.](https://www.ncbi.nlm.nih.gov/pubmed/16447487) 2005;71(12):1082-1085.  16. Crippa S, Bonardi C, Bovo G, et al. Pancreaticoduodenectomy for Pancreatic Metastases from Breast Carcinoma. J Pancreas (Online) 2004; 5(5):377-383.  17. Kitamuraj N, Murata S, Abe H, et al. Obstructive jaundice in a metastatic tumor of the pancreas from breast cancer: a case report. Jpn J Clin Oncol. 2003; 33(2): 93-97.  18. Nomizu T, Katagata N, Matsuoka T, et al. A case of breast cancer metastatic to the head of the pancreas. Breast Cancer. 1999; 6 (2): 131-134.  19. Z’graggen K, Fernández-del Castillo C, Rattner DW, et al. Metastases to the pancreas and their surgical extirpation. Arch Surg. 1998; 133 (4): 413-417.  20. Mountney J, Maury AC, Jackson AM, et al. Pancreatic metastases from breast cancer: an unusual cause of biliary obstruction. Eur J Surg Oncol. 1997; 23 (6): 574-576.  21. Titus AS, Baron TH, Listinsky CM, et al: Solitary breast metastasis to the ampulla and distal common bile duct. Am Surg. 1997; 63(6):512–515.  22. Papo M, Fernandez J, Quer JC, et al: Metastatic breast carcinoma presenting as obstructive jaundice. Am J Gastroenterol. 1996; 91(10):2240–2241. \|  \| \| --- \| --- \| \|  \|  \| |
| --- | --- | --- | --- | --- |
